# Real-World Experience With Janus Kinase Inhibitors in Immune-Mediated Diseases: Clinical Experience of a University Hospital

**DOI:** 10.7759/cureus.67729

**Published:** 2024-08-25

**Authors:** Marco Aurelio Ramirez Huaranga, Luis Angel Calvo Pascual, David Velasco Sanchez, Lourdes Martin de la Sierra Lopez, Laura Jimenez Rodriguez, Alberto Lopez Menchero Mora, David Castro Corredor, Marina Gonzalez Peñas

**Affiliations:** 1 Department of Rheumatology, Hospital General Universitario de Ciudad Real, Ciudad Real, ESP; 2 Department of Quantitative Methods, Instituto Católico de Administración y Dirección de Empresas (ICADE), Comillas Pontifical University, Madrid, ESP

**Keywords:** real-world, disease activity, psoriatic arthritis, spondyloarthritis, rheumatoid arthritis, jak inhibitors

## Abstract

Background: Several Janus kinase (JAK) inhibitors have been developed in recent years. These agents are widely applicable in clinical practice as an alternative treatment for immune-mediated diseases. While the safety and efficacy profile of these drugs has been evaluated in several randomized clinical trials and studies, very few authors have assessed safety and effectiveness under the real-world conditions of daily clinical practice.

Objective: This study aims to describe the effectiveness and safety of JAK inhibitors in daily clinical practice for the treatment of immune-mediated rheumatic diseases in a university hospital.

Methods: We performed a single-center observational, descriptive, retrospective study of all patients with rheumatoid arthritis (RA), spondyloarthritis (SpA), and psoriatic arthritis (PsA) receiving active treatment with JAK inhibitors between March 2022 and February 2023. We recorded study variables from the clinical history for subsequent analysis using STATA 12.0 (StataCorp LLC, College Station, TX). A 95% confidence interval was applied.

Results: The final analysis was performed on 64 patients (upadacitinib: 27, baricitinib: 16, tofacitinib: 13, filgotinib: eight), with a mean age of 55.69±10.78 years (60.94% females). The distribution by disease was as follows: RA, 44 (70.31%); SpA, 11 (17.18%); and PsA, eight (12.5%). A significant improvement was observed in all groups at six to 12 months, as follows: RA, remission in 48.89% and low activity in 26.67%; SpA, remission in 9.09% and low activity in 54.54%; and PsA, low activity in 87.5%. The factors most associated with poor response to treatment were activity before initiation of treatment and previous failure of biological disease-modifying antirheumatic drugs (bDMARDs). Adverse effects and complications were detected in 26.56% (SARS-CoV-2, one case; basal cell carcinoma, one case; and herpes zoster, two cases). There were no reports of cardiovascular or thromboembolic events, opportunistic infection, or tuberculosis.

Conclusions: Our real-world data show that treatment with JAK inhibitors leads to a high rate of remission/low activity that remains unchanged at six to 12 months in RA, SpA, and PsA. The predictors of a poor response to JAK inhibitors in our study population were the level of activity before initiation of treatment and previous failure of bDMARDs. No cardiovascular or thromboembolic events were reported. Of note, we did record one case of severe infection, one case of basal cell carcinoma, and two cases of herpes zoster.

## Introduction

Janus kinase (JAK) refers to a group of enzymes in the cell cytoplasm that exert tyrosine kinase activity to facilitate the transmission of signals from the cell surface to its interior. Various proinflammatory cytokines use this pathway for intracellular signaling [1].

Recent years have seen the development of several JAK inhibitors that have proven widely applicable in clinical practice as an alternative treatment for immune-mediated diseases. The first JAK inhibitors approved for the treatment of rheumatoid arthritis (RA) were tofacitinib and baricitinib, whose indications were subsequently extended to the treatment of other autoimmune diseases. Newer JAK inhibitors include upadacitinib and filgotinib, which, after completion of their various development phases, have eventually been approved for immune-mediated rheumatic diseases [2,3]. These drugs have a safety and adverse effects profile similar to that of traditional biologics (e.g., anti-tumor necrosis factor (TNF), anti-interleukin (IL)-17, and anti-IL-6 agents). However, reactivation of the herpes zoster virus and thromboembolic events seem to be more common in patients treated with JAK inhibitors [4].

Tofacitinib was the first JAK inhibitor to be approved (JAK1/JAK3, with some activity against JAK2) for the treatment of autoimmune diseases. It is therefore the drug for which most information is available. Study results show tofacitinib to be both safe and efficacious when combined with other conventional synthetic disease-modifying antirheumatic drugs (csDMARDs) for the treatment of RA, spondyloarthritis (SpA), and psoriatic arthritis (PsA) [5, 6]. Baricitinib is a selective JAK1/JAK2 inhibitor. Several phase III studies have shown its efficacy in RA [7, 8]. Upadacitinib is a new-generation JAK inhibitor that is 74 times more selective for JAK1 than for JAK2 and is therefore considered more specific for JAK1. Two multicenter randomized, double-blind, placebo-controlled phase II trials have been performed in patients with moderate-to-severe RA not responding to anti-TNF agents or methotrexate. Both studies showed a rapid improvement for upadacitinib compared with placebo in terms of the American College of Rheumatology 20/50/70 response criteria and the Disease Activity Score in 28 joints (DAS28) with C-reactive protein (CRP). Upadacitinib is currently indicated for RA, SpA, and PsA [9, 10]. Filgotinib inhibits both JAK1 and JAK2. However, it is 30 times more selective for JAK1. Studies in patients with active RA and an inadequate response to methotrexate showed the efficacy of filgotinib in monotherapy over placebo. This finding was confirmed in 2 phase IIb trials, DARWIN1 and DARWIN2 [11, 12].

While several studies and randomized clinical trials have evaluated the safety and efficacy profile, very few have studied the effectiveness and safety of these drugs under real-world conditions of clinical practice. One such study was recently published by González et al. [13], who analyzed the real-world effectiveness and safety of tofacitinib and baricitinib in 98 patients with RA. The authors observed a significant reduction in the activity indices of both treatment arms, with no clear differences between them. Low activity was achieved in 30% and remission in 13% at three months, with adequate disease control in 64% at six months. Adverse events were reported in 55%, although only 18% were relevant. Of these, we highlight pneumonia (6%) and herpes zoster infection (3%). There were no reports of thromboembolic events, active tuberculosis, neoplasm, or other major cardiovascular events. Treatment was discontinued in 30% of patients owing to primary failure (4%), secondary failure (4%), and adverse events (11%).

Consequently, the objective of our study was to report on the real-world safety and effectiveness of JAK inhibitors in patients with immune-mediated rheumatic diseases treated in a university hospital.

## Materials and methods

We performed a single-center retrospective, descriptive, observational study of all patients with RA, SpA, and PsA (based on the classification criteria of the European Alliance of Associations for Rheumatology (EULAR) [14], Assessment of SpondyloArthritis International Society (ASAS) [15], and Classification for Psoriatic Arthritis (CASPAR) [16]), respectively) who were receiving treatment with JAK inhibitors (tofacitinib, baricitinib, upadacitinib, and filgotinib) between March 2022 and February 2023. We excluded patients whose clinical history did not include the study variables and patients with off-label indications. The protocol was approved by the Research and Ethics Committee of Integrated Care Management, Ciudad Real, Spain (Registry 01/2023), and all patients gave their informed consent to participate.

We retrieved information on the study variables from the clinical history. We recorded age, sex, cardiovascular risk factors, diagnoses, time since diagnosis, serologic markers, baseline disease activity, type of JAK inhibitor, concomitant treatment, previous treatment, time receiving JAK inhibitors, disease activity during follow-up, and adverse events.

The information collected was entered into the study database. Quantitative variables were expressed as mean and standard deviation; qualitative variables were expressed as numbers and percentages. Data were analyzed by comparing the means (t-test) for erythrocyte sedimentation rate (ESR), CRP, DAS28-CRP, the Ankylosing Spondylitis Disease Activity Score with CRP (ASDAS-CRP), and the Disease Activity Index for PsA in 28 joints (DAPSA28). The efficacy of JAK inhibitors was assessed using means and quartiles by contrasting hypotheses on the level of activity (DAS28-CRP, ASDAS-CRP, and DAPSA28), the difference in means, and boxplots. Finally, the variables with the greatest effect on “no efficacy (poor response)” were identified using the mutual information test with the Scikit-learn (sklearn) Library (Scikit-learn: Machine Learning in Python, Pedregosa et al., JMLR 12, pp. 2825-2830, 2011). Compared with classic tests such as the chi-squared test or F-test, the mutual information test detects nonlinear associations using the Kullback-Leibler divergence to capture the discrepancy between two probability distributions. To ensure a robust estimation, we performed the test 1,000 times and averaged the results. All analyses were performed with a 95% confidence interval using STATA 12.0 (StataCorp LLC, College Station, TX).

## Results

Of a total of 69 patients, five were excluded owing to incomplete data and/or off-label indications (tofacitinib: one, baricitinib: two, and upadacitinib: two). The final analysis was based on 64 patients (upadacitinib: 27, baricitinib: 16, tofacitinib: 13, and filgotinib: eight) with a mean age of 55.69±10.78 years (60.94% female). 

Our cohort had no history of cardiovascular or thrombotic disease, although patients did have cardiovascular risk factors (e.g., arterial hypertension: 31.25%; dyslipidemia: 26.56%; current smoking: 14%; diabetes: 4.68%). Disease was distributed as follows: RA, 44 cases (70.31%) with a time since diagnosis of 10.53±6.58 years; SpA, 11 cases (17.18%) with a time since diagnosis of 10.55±7.08 years; and PsA, eight cases (12.5%) with a time since diagnosis of 8±5.07 years. Serologic characteristics, concomitant treatment, and failure of previous treatment are shown in Table 1.

**Table 1:** Serologic characteristics, concomitant treatment, and treatment before the initiation of JAK inhibitors JAK: Janus kinase; N: number; SD: standard deviation; RF: rheumatoid factor; ACPA: anti-citrullinated peptide antibody; HLA: human leukocyte antigen; NSAID: nonsteroidal anti-inflammatory drug; DMARD: disease-modifying antirheumatic drug; csDMARD: conventional synthetic DMARD; TNF: tumor necrosis factor

Variables	N (%)/ mean (±SD)
Autoimmune serology results	
Rheumatoid arthritis (n=45)	
RF (+)	36 (56.25)
ACPA (+)	37 (57.81)
SpA and PsA (n=19)	
HLA-B27 (+)	13 (20.31)
Concomitant treatment	
Rheumatoid arthritis (n=45)	
NSAIDs	15 (33.33)
No corticosteroids	25 (55.55)
Corticosteroids <10 mg prednisone	20 (44.44)
Methotrexate	10 (22.22)
Leflunomide	3 (6.66)
Hydroxychloroquine	2 (4.44)
Spondyloarthritis (n=11)	
NSAIDs	11 (100)
No corticosteroids	10 (90.9)
Corticosteroids <10 mg prednisone	1 (9.1)
Psoriatic arthritis (n=8)	
NSAIDs	6 (75)
No corticosteroids	7 (87.5)
Corticosteroids <10 mg prednisone	1 (12.5)
Methotrexate	2 (25)
Leflunomide	2 (25)
Failure of previous treatment	
Rheumatoid arthritis (n=45)	
csDMARDs	38 (84.44)
1 Anti-TNF	37 (82.22)
2 Anti-TNF	13 (28.89)
3 Anti-TNF	7 (15.55)
Anti–IL-6	14 (31.11)
Rituximab	2 (4.44)
Abatacept	8 (17.78)
1 JAK inhibitor	13 (28.89)
2 JAK inhibitors	2 (4.44)
Spondyloarthritis (n=11)	
csDMARDs	6 (54.54)
1 Anti-TNF	11 (100)
2 Anti-TNF	7 (63.64)
3 Anti-TNF	2 (18.18)
Anti–IL-17	5 (45.45)
1 JAK inhibitor	1 (9.09)
Psoriatic arthritis (n=8)	
csDMARDs	7 (87.5)
1 Anti-TNF	7 (87.5)
2 Anti-TNF	3 (37.5)
3 Anti-TNF	0 (0)
Anti–IL-17	2 (25)
1 JAK inhibitor	1 (12.5)

**Figure 1:**
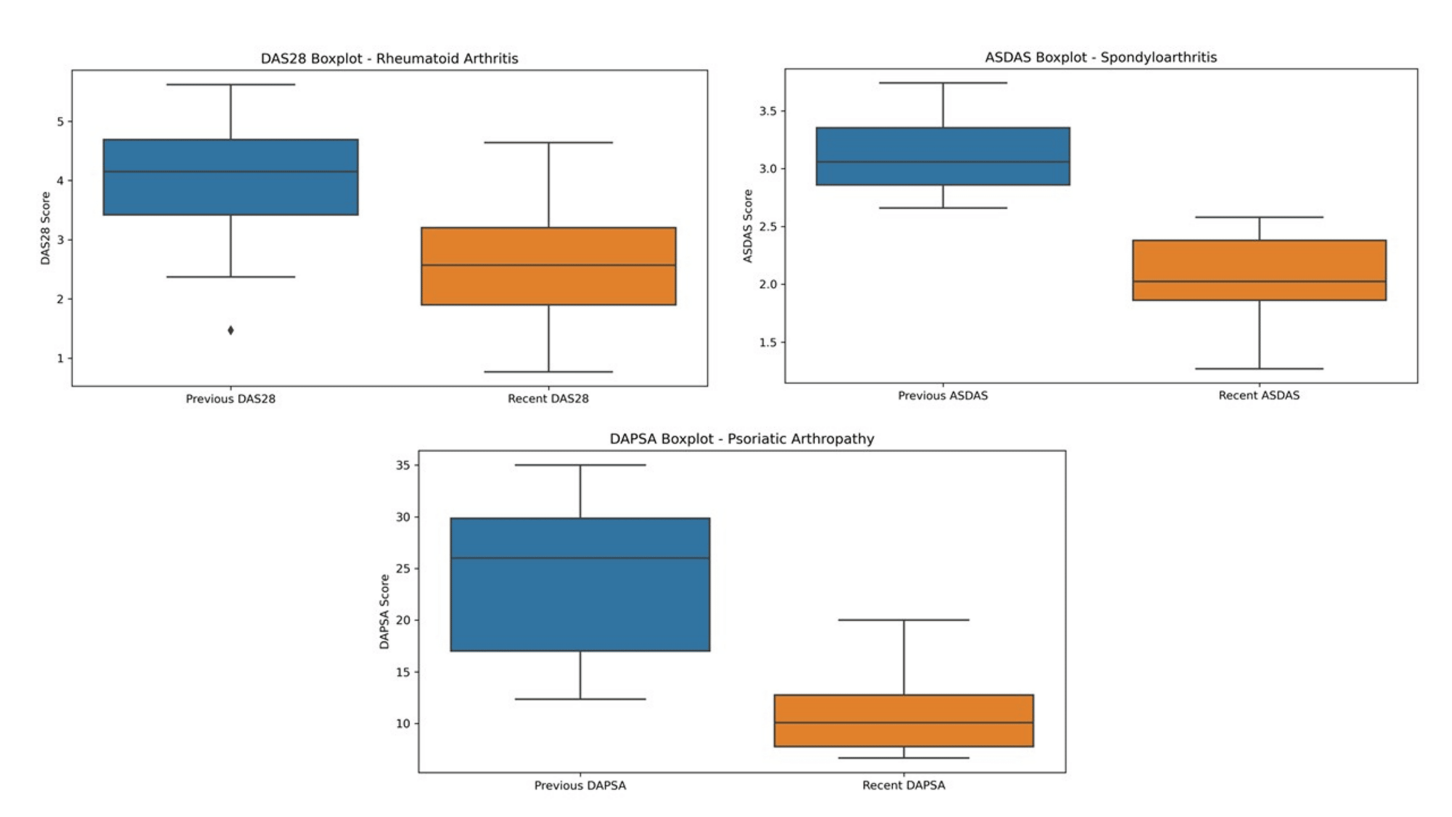
Boxplots showing the effectiveness analysis according to disease activity indices DAS28: Disease Activity Score in 28 Joints; ASDAS: Ankylosing Spondylitis Disease Activity Score; DAPSA: Disease Activity Index for Psoriatic Arthritis

**Figure 2:**
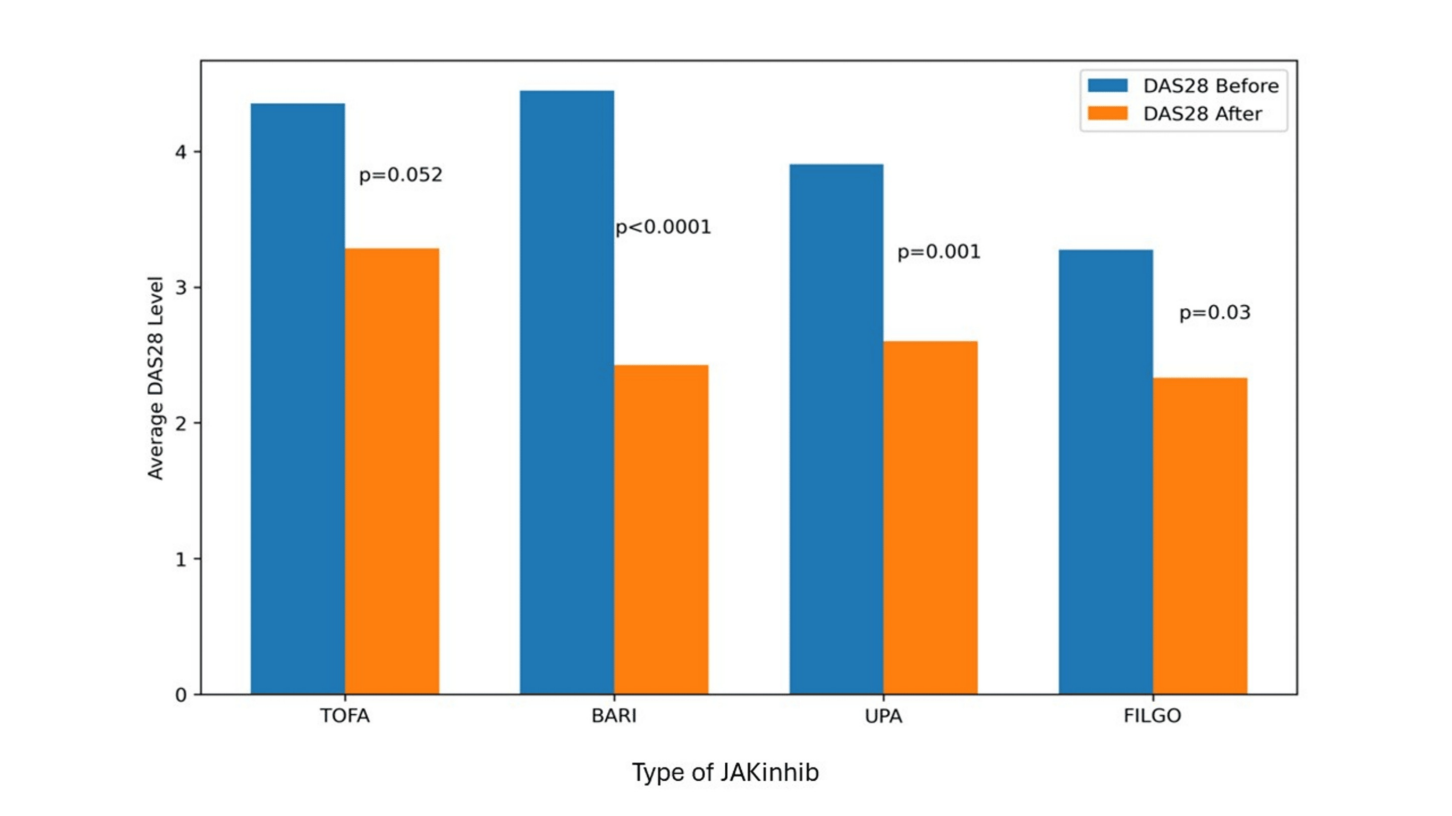
Variation in DAS28 scores after the initiation of JAK inhibitors by drugs in patients with rheumatoid arthritis DAS28: Disease Activity Score in 28 Joints; JAK: Janus kinase

Table 2 shows the response to treatment overall and by disease. Analysis of serologic markers only revealed a significant decrease in ESR overall (15.77 to 11.22 mm/h, p=0.004) and in RA (18.47 to 13.07 mm/h, p=0.008). As for disease activity indices (DAS28, ASDAS, and DAPSA28), a significant improvement was observed in all three, as shown in the boxplot analysis (Figure 1). In the RA group, 48.89% of patients achieved remission and 26.67% achieved low activity. In the SpA group, 9.09% achieved remission, and 54.54% achieved low activity. In the PsA group, 87.5% achieved low disease activity. One interesting observation in the RA group was that when the DAS28 was analyzed by drug, the disease seemed to be better controlled with baricitinib, even though the drug was started with higher levels of disease activity (Figure 2).

**Table 2:** Disease activity at the initiation of treatment with JAK inhibitors and during follow-up JAK: Janus kinase; N: number; SD: standard deviation; RF: rheumatoid factor; ESR: erythrocyte sedimentation rate; CRP: C-reactive protein; DAS28: Disease Activity Score-28; ASDAS: Ankylosing Spondylitis Disease Activity Score; DAPSA: Disease Activity Index for Psoriatic Arthritis

	Baseline	Follow-up (6-8 months)
	N (%) / mean (±SD)	N (%) / mean (±SD)
ESR (mm/h)	15.77±16.91	11.22±10.16
CRP (mg/dl)	0.69±0.94	0.46±0.8
Rheumatoid arthritis		
ESR (mm/h)	18.47±18.31	13.07±11.24
CRP (mg/dl)	0.8±1.04	0.55±0.93
DAS28	4.07±0.89	2.62±0.92
Remission (≤2.6)	3 (6.66)	22 (48.89)
Low activity (>2.6-3.2)	2 (4.44)	12 (26.67)
Moderate activity (>3.2-5.1)	33 (73.33)	11 (24.44)
High activity (>5.1)	7 (15.55)	0 (0)
Spondyloarthritis		
ESR (mm/h)	9.45±11.39	8.82±5.31
CRP (mg/dl)	0.32±0.24	0.32±0.37
ASDAS	3.08±0.4	2.05±0.4
Inactive (< 1.3)	0 (0)	1 (9.09)
Low activity (<2.1)	0 (0)	6 (54.54)
High activity (≤3.5)	9 (81.82)	4 (36.37)
Very high activity (>3.5)	2 (18.18)	0 (0)
Psoriatic arthritis		
ESR (mm/h)	9.25±11.11	4.13±2.36
CRP (mg/dl)	0.55±0.81	0.18±0.15
DAPSA28	24.47±8.36	11.1±4.5
Remission (≤4)	0 (0)	0 (0)
Low activity (>4 to ≤14)	1 (12.5)	7 (87.5)
Moderate activity (>14 to ≤28)	4 (50)	1 (12.5)
Very high activity (>28)	3 (37.5)	0 (0)

**Figure 3:**
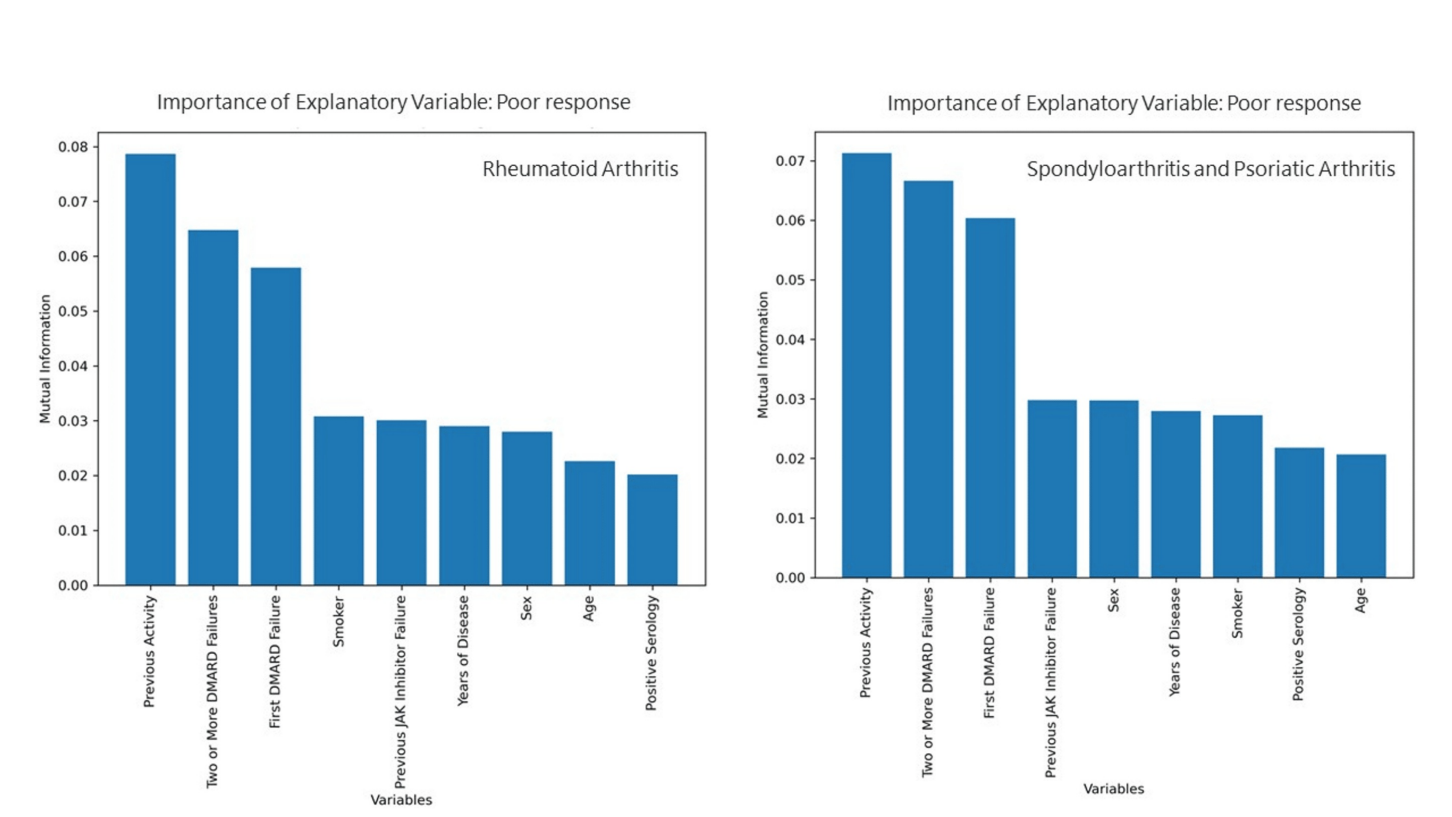
Effect of clinical variables by disease as predictors of poor response to JAK inhibitors JAK: Janus kinase; DMARDs: disease-modifying antirheumatic drugs

The mutual information test was performed to analyze the influence of clinical and serologic variables as predictors of a poor response to JAK inhibitors. In the RA group, previous activity and failure of biological disease-modifying antirheumatic drugs (bDMARDs) were clearly the major determinants, whereas in the SpA and PsA groups, the only notable finding was for the level of disease activity before initiation of treatment (Figure 3).

During follow-up, 26.56% of patients experienced an adverse effect or complication, most of which were mild and transient. We recorded only one case of severe infection (bilateral pneumonia caused by SARS-CoV-2), one case of nonmelanoma skin cancer (basal cell carcinoma), and no cardiovascular or thromboembolic events, opportunistic infections, or tuberculosis. Mild upper respiratory tract infections (pharyngitis/pharyngotonsillitis) were recorded in four patients with RA and in four with SpA. There were only two cases of herpes zoster infection in the RA group and elevated transaminase values in three patients with RA and in one with PsA. Lastly, we recorded a case of interstitial pneumonia and a case of acneiform eruption.

## Discussion

Data on the real-world use of JAK inhibitors are increasingly published, especially those used to treat RA. A review of publications on the real-world use of tofacitinib in RA between 2018 and 2020 revealed one-year remission to be 48%-53% according to the DAS28. Similarly, the persistence of treatment was similar to that of bDMARDs, and the frequency of adverse effects was similar to that reported in clinical trials [17]. A recently published retrospective study (2024) of 122 patients with RA treated with tofacitinib found a significant improvement (p<0.0001) in the DAS28-CRP, Clinical Disease Activity Index, and Simplified Disease Activity Index at three, six, and 12 months of follow-up, with a persistence rate of 89.35%. Two major cardiovascular events-a case of herpes zoster and a case of lymphoma-were also reported [18]. In a retrospective study of 182 patients receiving baricitinib in Spain (100% had received csDMARDs, 78% had received bDMARDs, and 43.4% were receiving baricitinib in monotherapy) and followed up for approximately six to 12 months, the authors found that 71.6% achieved remission or low activity at six months and that 76.3% reached these goals at 12 months [19]. A systematic review of publications on real-world use of baricitinib in RA observed that in most cases, the drug was used after failure of bDMARDs, with remission/low activity reached by 60% of patients at 12 weeks and 81.6% at 24 weeks [20]. A 2024 Canadian study of 392 patients with RA receiving upadacitinib found that 63.5% achieved remission at six months [21]. Similarly, a 2024 real-world study of filgotinib administered to 126 patients with RA revealed an improvement in symptoms at six months (patient global assessment, examiner global assessment, visual analog scale, DAS28-CRP, and CRP), with low activity in 37.2% and remission in 10.7%. The response was better in the bDMARD-naive group. The authors reported only one major cardiovascular event and one case of transient hypertransaminasemia [22]. Finally, an Italian study from the same year reported on real-world experience with JAK inhibitors in 115 patients with RA (17 baricitinib, 32 filgotinib, 21 tofacitinib, and 45 upadacitinib) after evaluating the clinical response at three, six, and 12 months of follow-up. All four drugs were associated with a clear improvement in the DAS28 from the third month onward; this persisted until 12 months (p = 0.0001), with no differences between the drugs. There was only one report of a thrombotic event and a major cardiovascular event in patients treated with baricitinib, a case of herpes zoster in those treated with filgotinib and tofacitinib, and a case of nonmelanoma skin cancer in patients taking upadacitinib [23]. Another real-world study on the safety of tofacitinib, baricitinib, upadacitinib, and filgotinib in RA reported the frequency of adverse events to be 18%, with the most notable findings being one cerebrovascular accident and three deaths associated with severe infection. There were no differences between the drugs used [24]. We obtained similar results in patients with RA, with control of disease in 75.56% (remission and low activity according to DAS28-CRP).

Real-world studies on SpA and PsA are scarce. A retrospective study from India on the use of tofacitinib in 100 patients with SpA reported significantly improved disease activity values (ASDAS-CRP) after six months of follow-up [25]. Another recent study of the effectiveness of tofacitinib and adalimumab in axial SpA found low disease activity (ASDAS) at four months of treatment; this was greater in the tofacitinib group (71.6% vs. 47.9%) [26]. In our study, 63.63% of patients with SpA achieved remission/low activity (ASDAS-CRP).

As for PsA, a 2022 study of 318 patients receiving tofacitinib revealed that at six months, 71.1% were still receiving treatment owing to remission/low activity [27]. An Australian study of 406 PsA patients receiving tofacitinib showed that the drug was used as first-line treatment in only 19.2% of cases, although patients were still receiving treatment at 16.5 months, thus leading the drug to be considered effective in the long term [28]. In our study, 87.5% of patients with PsA achieved low activity (DAPSA28). 

Although the results demonstrated good effectiveness of the JAK inhibitors in immune-mediated diseases, it is important to note that this study is descriptive and has a small sample. Therefore, we cannot generalize our findings. However, the results of our cohort are similar to those described by studies with a more complex design and a larger number of patients. These findings provide a foundation upon which we can design prospective, randomized, and controlled studies. 

## Conclusions

In conclusion, our real-world findings show that JAK inhibitors frequently achieve remission/low activity that is maintained for up to six to 12 months in patients with RA, SpA, and PsA. The predictors of poor response to JAK inhibitors in our study population were the level of disease activity before initiation of treatment and previous failure of bDMARDs. As for safety, while it is necessary to consider the recommendations of the Pharmacovigilance Risk Assessment Committee of the European Medicines Agency, we observed that adverse events are clearly less frequent than expected both in our cohort and in the literature.
